# Fluctuating selection in a monkeyflower hybrid zone

**DOI:** 10.1093/evlett/qrae050

**Published:** 2024-09-26

**Authors:** Diana Tataru, Max De Leon, Spencer Dutton, Fidel Machado Perez, Alexander Rendahl, Kathleen G Ferris

**Affiliations:** Department of Ecology and Evolutionary Biology, Tulane University, New Orleans, LA, United States; Department of Ecology and Evolutionary Biology, Tulane University, New Orleans, LA, United States; Department of Ecology and Evolutionary Biology, Tulane University, New Orleans, LA, United States; Department of Ecology and Evolutionary Biology, Tulane University, New Orleans, LA, United States; Life & Environmental Sciences Department, University of California Merced, Merced, CA, United States; Department of Ecology and Evolutionary Biology, Tulane University, New Orleans, LA, United States; Department of Ecology and Evolutionary Biology, Tulane University, New Orleans, LA, United States

**Keywords:** hybridization, natural selection, adaptation

## Abstract

While hybridization was viewed as a hindrance to adaptation and speciation by early evolutionary biologists, recent studies have demonstrated the importance of hybridization in facilitating evolutionary processes. However, it is still not well-known what role spatial and temporal variation in natural selection play in the maintenance of naturally occurring hybrid zones. To identify whether hybridization is adaptive between two closely related monkeyflower species, *Mimulus guttatus* and *Mimulus laciniatus*, we performed repeated reciprocal transplants between natural hybrid and pure species’ populations. We planted parental genotypes along with multiple experimental hybrid generations in a dry (2021) and extremely wet (2023) year in the Sierra Nevada, CA. By taking fine-scale environmental measurements, we found that the environment of the hybrid zone is more similar to *M. laciniatus’s* seasonally dry rocky outcrop habitat than *M. guttatus’s* moist meadows. In our transplants hybridization does not appear to be maintained by a consistent fitness advantage of hybrids over parental species in hybrid zones, but rather a lack of strong selection against hybrids. We also found higher fitness of the drought-adapted species, *M. laciniatus,* than *M. guttatus* in both species’ habitats, as well as phenotypic selection for *M. laciniatus-*like traits in the hybrid habitat in the dry year of our experiment. These findings suggest that in this system, hybridization might function to introduce drought-adapted traits and genes from *M. laciniatus* into *M. guttatus*, specifically in years with limited soil moisture. However, we also find evidence of genetic incompatibilities in second generation hybrids in the wetter year, which may balance a selective advantage of *M. laciniatus* introgression. Therefore, we find that hybridization in this system is both potentially adaptive and costly, and that the interaction of positive and negative selection likely determines patterns of gene flow between these *Mimulus* species.

## Introduction

Hybridization, or gene flow between species, was seen as a barrier to speciation and adaptation among early taxonomists and speciation researchers (Arnold, 1997; Barton & Hewitt, 1985). The contribution of gametes to unfit offspring complicated ideas of natural selection introduced by Darwin and muddled the definition of species as groups of reproductively isolated populations (Coyne & Orr, 2004; Mayr, 1942). This “problem with hybridization” was further corroborated by the discovery of coadapted gene complexes within species and genetic incompatibilities between species that lead to decreased fitness upon secondary contact. Intrinsic genetic incompatibilities are often masked in first-generation hybrids due to heterozygosity, but negative epistatic interactions between alleles are expressed in later hybrid generations (Bateson, 1909; Dobzhansky, 1936; Muller, 1942). Low hybrid fitness in secondary contact could also be due to genotype × environment (G × E) interactions causing extrinsic post-zygotic reproductive isolation (Grant V., 1966; Hatfield & Schluter, 1999). While many of these factors do restrict hybridization, evolutionary biologists continue to discover evidence of interspecific gene flow across taxa (Abbott et al., 2013; Grant & Grant, 2016; Moran et al., 2021; Sianta et al., 2024).

Hybridization was first described as a mechanism of rapid evolution and eventual speciation in the context of adaptation to disturbed habitats (Anderson & Stebbins, 1954; Schemske, 2000). More recent genomic studies have uncovered hybridization across taxa and habitats as a source of speciation and adaptation through accelerated introgression, increased genetic variation, and heterozygote advantage (Abbott et al., 2013; Coyne & Orr, 2004; Kim & Rieseberg, 1999; Mitchell et al., 2019; Stebbins, 1959). One prevailing hypothesis for adaptive introgression is that hybridization is maintained by high fitness in intermediate environments and against hybrids in parental habitats, forming a cline across environmental gradients (Moore, 1977). This has been identified in natural systems such as *Artemisia tridentata,* with higher hybrid fitness at an intermediate elevation (Miglia et al., 2005; Wang et al., 1997), and in Darwin’s finches, with selection for intermediate beak morphology (Grant & Grant, 1996, 2016). Testing these patterns with experimental hybridization in the wild rather than correlative studies can distinguish between the evolutionary forces that shape patterns of introgression between species (Hatfield & Schluter, 1999; Lexer et al., 2003; Moran et al., 2021).

Identifying the role of natural selection in shaping population structure is key to understanding the maintenance of hybridization in the wild. Rather than being a consistently directional force pushing populations to an optimum, selection can vary greatly on a spatial and temporal scale (Siepielski et al., 2013; Tataru et al., 2023; Wadgymar et al., 2017). Genetic variation introduced by hybridization can facilitate the persistence of populations in the face of changing conditions, particularly at species’ range edges and during sudden intense environmental changes (Brasier, 1995; Grant & Grant, 2016). Tension zones are areas where hybridization is maintained by spatial and/or temporal variation in selection, specifically dispersal-selection balance (Bartoń, 2009; Gay et al., 2008; Key, 1968; Smith et al., 2013). When variable environmental conditions interact with novel genetic combinations to produce hybrids with superior fitness to their parental species, this is more aligned with the hybrid novelty model (Arnold, 1997). This environment-dependent hybrid advantage, where hybridization is maintained by G × E interactions, is well-documented in Louisiana *Iris* species (Johnston et al., 2001). Identifying the role of environmental variation in shaping hybridization is critical to gaining a deeper understanding of species’ persistence in a changing world.

**Figure 1.**
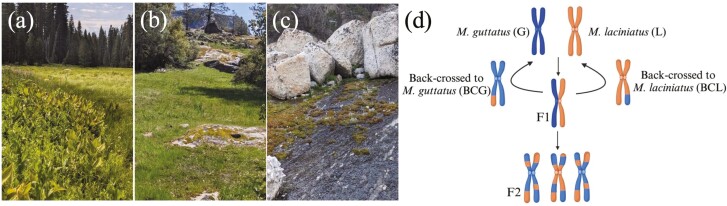
Pictures of (A) *M. guttatus* parental habitat at the end of the season (B) hybrid zone habitat (C) experimental plot set-up in *M. laciniatus* parental habitat, and (D) crossing design of six genotypes, with colors indicating parental genetic material (created with Biorender.com).

To test whether hybridization is adaptive in natural hybrid zones, we compared two closely related species in the *Mimulus guttatus* (syn. *Erythranthe guttata*) species complex that hybridize in sympatry (Vickery, 1964). We conducted reciprocal transplants in hybrid and parental habitats in Yosemite National Park, CA using *M. guttatus, Mimulus laciniatus* (syn. *Erythranthe laciniata*), F_1_, F_2_, and reciprocally backcrossed hybrids (Figure 1). We measured phenotypic selection on traits previously identified to be under divergent selection between parental species’ habitats (Ferris & Willis, 2018; Tataru et al., 2023) and fitness differences in two years with drastically different water availabilities. The second year of the experiment (2023) experienced three times the average amount of snowpack, creating extreme conditions and episodic selection. In California’s Mediterranean climate, snowpack and the subsequent amount of snowmelt are critical to water availability in both species’ habitats. In the rocky outcrop habitat of *M. laciniatus* snowmelt is ephemeral and dries up quickly, while the deeper soils of *M. guttatus’s* meadows dry out gradually throughout the growing season (Ferris &Willis, 2018; Ferris et al., 2014; Tataru et al., 2023). Natural hybrid zones seem to span intermediate habitats, with both meadow and rocky outcrop microhabitats present (Ferris et al., 2014; Tataru, personal observation).

In our reciprocal transplant experiments we replicated various stages of hybridization likely to be present in a natural hybrid population. Asymmetric introgression due to more frequent backcrossing into one parental species has been well-documented across self-fertilizing vs. outcrossing species pairs similar to *M. laciniatus* (selfer) and *M. guttatus* (outcrosser) (Brandvain et al., 2014; Ruhsam et al., 2011; Sianta et al., 2024). We can test for evidence of adaptive asymmetric introgression by comparing the relative fitness of each direction of experimental backcross hybrids. To our knowledge, there are relatively few experimental hybridization studies of this scale that empirically examine the interaction of natural selection and gene flow in naturally hybridizing species (but see Campbell et al., 2024; Mitchell et al., 2019). Using repeated reciprocal field transplants and fine-scale environmental measurements, we aim to answer the following questions: (a) Is the hybrid zone more ecologically similar to the habitat of one species or another? (b) Is gene flow between species advantageous in natural *Mimulus* hybrid zones? (c) How does temporally fluctuating selection influence the fitness of hybrids? (d) Which traits are advantageous and under selection in hybrid versus parental habitats?

## Methods

### General reciprocal transplant design

To investigate whether hybridization is adaptive in natural hybrid zones of *M. laciniatus* and *M. guttatus*, we conducted reciprocal transplants in Yosemite National Park, CA, USA over two years with contrasting snowpack levels. We chose transplant sites by the presence of parental species or hybrids. The hybrid zone (Figure 1B, Lat: 37.957206, Long: −119.78607, Elevation: 1,207 m) is a moist meadow surrounded by rocky habitat where *M. guttatus*, *M. laciniatus,* and natural hybrids co-occur. The *M. guttatus* site (Figure 1A, Lat: 37.756032, Long: −119.803024, Elevation: 1,841 m) is a mesic meadow where native *M. guttatus* grows along a seep. Finally, the *M. laciniatus* site (Figure 1C, Lat: 37.85241103, Long: −119.441278, Elevation: 2,562 m) is a granite outcrop with native *M. laciniatus* growing on shallow rocky soils and moss fed by ephemeral snowmelt. The first year of the transplant, 2021, was a drought year with 59% April 1st average snowpack in the Sierra Nevada, CA (CDEC, 2024). Experiments ran from April 11th–August 2nd, 2021. The second year, 2023, experienced 245% average snowpack (CDEC, 2024) and experiments ran May 7th–November 14th.

### Environmental variation within and between habitats

To determine environmental variation among our hybrid and parental sites, we took fine-scale environmental measurements at each site across the growing season in both years. We measured soil moisture with a Dynamax SM150 Soil Moisture sensor, surface temperature using a laser thermometer, and light measurements with an Apogee MQ-200X Sunlight Quantum Meter at each block and site every week. We conducted all analyses in R Statistical Software (v4.2.1, R Core Team, 2022). To test the effect of environmental variables on survival we used linear mixed effects models in the R package *nlme* (Pinheiro et al., 2023) with plant survival as the dependent variable, and soil moisture, surface temperature, light levels, date, and site as dependent variables, and block as a random effect. We used the R package *MuMin* to conduct model selection (Bartoń, 2009). We measured survival as the proportion of plants surviving in each block at the time of each environmental measurement.

To test whether the hybrid zone was either environmentally intermediate or more similar to one of the parental species’ sites, we tested for differences between the slopes of soil moisture decrease over time by running linear mixed effects models in *nlme* (Pinheiro et al., 2023) with soil moisture as a dependent variable and site, time, and their interaction as independent variables. We then used the R package *emmeans* to compare slopes of linear regressions (Lenth, 2016). To quantify differences in the shape of seasonal soil moisture curves, we ran principal components analyses (PCAs) on each year separately. Using the method outlined in Cuthill et al. (1999) we binned measurements by week and input each week as a variable in the PCA. PC1 represents overall variation in total soil moisture values, while PC2 and PC3 represent differences in shape of soil moisture curves over time. We conducted ANOVAs with post-hoc Tukey tests on PC axes (dependent variable) with site and block as independent variables.

### 2021 reciprocal transplant

**Figure 2.**
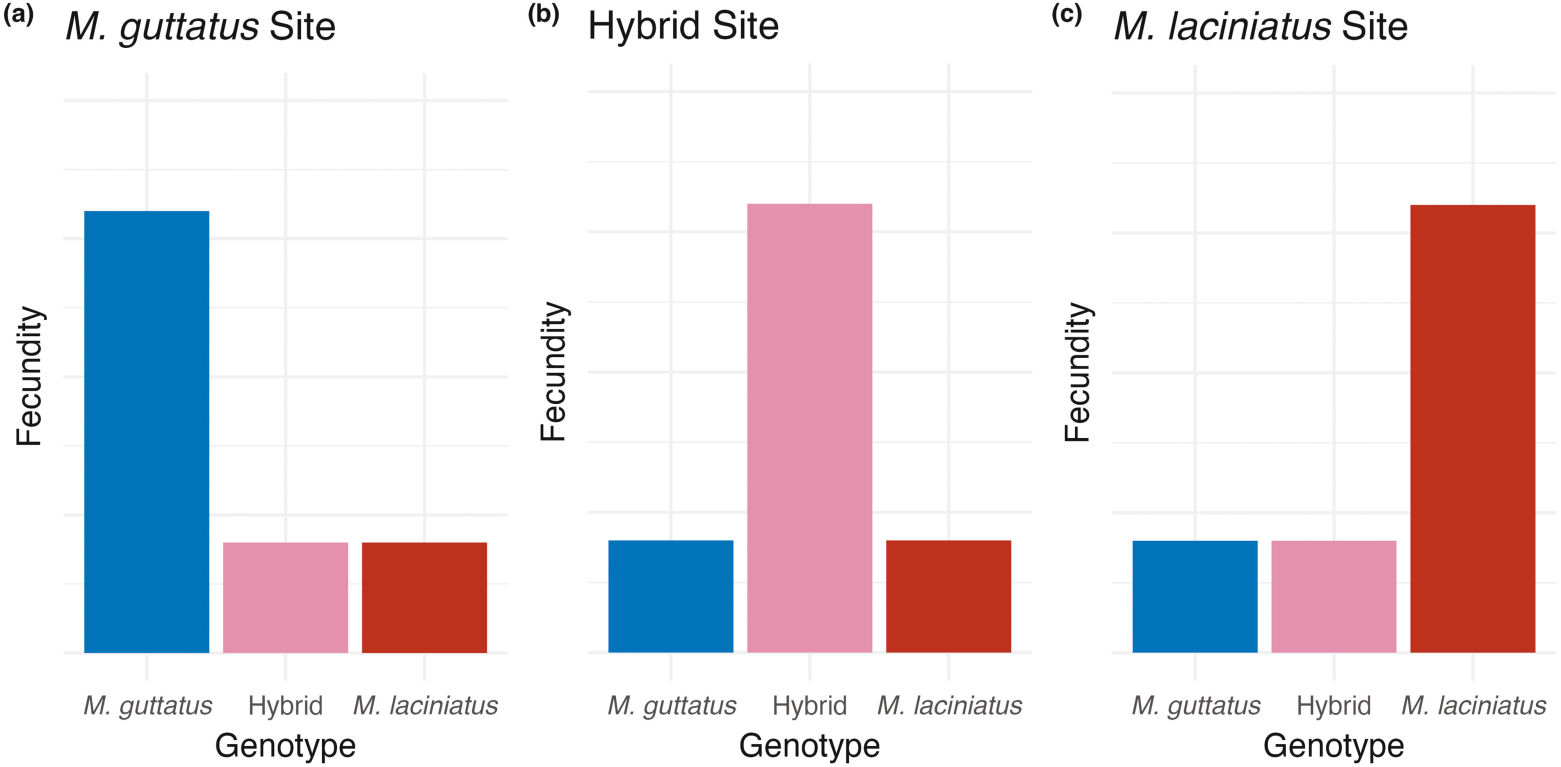
Predicted fecundity in each site given hypothesized fitness advantage of hybrids in hybrid sites, and local adaptation of each species in its native habitat. Plant genotypes are grouped by site, and genotype is indicated by color. Predictions show higher fitness of individuals with *M. guttatus* genes in the *M. guttatus* habitat (A), higher hybrid fitness in the hybrid habitat (B), and higher fitness of individuals with *M. laciniatus* genes in the *M. laciniatus* habitat (C).

To identify if hybrids have a fitness advantage in hybrid zones, we conducted a reciprocal transplant of parental species, F_1_, F_2_, and back-crossed hybrids in hybrid and parental habitats. We reciprocally crossed 25 genotypes of field collected *M. guttatus* (HG) and *M. laciniatus* (HL) from the natural hybrid population to make 50 unique F_1_ crosses. Field collected species were identified by divergent phenotypic traits between species and grown out for one generation in the greenhouse to confirm species identification. We backcrossed and self-fertilized each F_1_ to create backcross-*M. guttatus* (BCG), backcross-*M. laciniatus* (BCL), and F_2_ hybrids. We used these six genotypic categories (see Figure 1D) in a reciprocal transplant experiment in *M. guttatus*, *M. laciniatus*, and hybrid environments to test whether hybrids have a fitness advantage in the hybrid zone, and decreased fitness in parental environments (Figure 2). We staggered the timing of planting and transplanting so that our experimental seedlings would be at the same developmental stage as native *Mimulus* in each site (hybrid site April 11th; *M. guttatus* site April 28th; *M. laciniatus* site May 26th). We stratified soaked flats with seeds at 4°C for 14 days, and then germinated plants for one week in growth chambers at University of California, Davis. We transplanted seedlings at the cotyledon stage into 100 randomized blocks of 36 plants each (six per genotype) at each field site. Plants were approximately one inch apart and native *Mimulus* was removed from blocks. Due to limitations with germination, the total number of individuals at each site varied (Supplementary Table S1; Hybrid: 3,498 total, *M. guttatus*: 3,372 total, *M. laciniatus*: 2,358 total). To account for transplant shock, we replaced any individuals that died three days after planting.

We monitored sites every three days for timing of first flower, plant height, stigma-anther separation, flower width, leaf measurements, and herbivory. We measured herbivory as presence or absence of damage and analyzed differences in herbivory between years, sites, and genotypes and its effect on fitness (survival and fecundity), using a two-way ANOVA. We collected first true leaves on the day of first flower, digitally scanned them, and measured leaf area and lobing index through the program Image J (described in Ferris et al., 2015). Once plants senesced, we collected all fruits and counted total seed number for each plant, which we used as our lifetime fitness metric. We calculated average genotypic fecundity as the total seed number divided by the total number of plants planted for each genotype in each habitat.

### 2023 field transplant

To examine the role that temporal variation in selection plays in this system, we performed another reciprocal transplant in 2023. We added genotypes from each species’ specific habitat of *M. guttatus* and *M. laciniatus* in addition to the parents of our experimental crosses. In 2023, hybrids were created from one inbred genotype of each parent also used in the 2021 crossing design (*M. laciniatus* maternal × *M. guttatus* paternal). Parental genotypes were outcrossed to control for inbreeding depression (Hall & Willis, 2006). We created eight experimental genotypes: Hybrid zone *M. guttatus* (HG), Hybrid zone *M. laciniatus* (HL), *M. guttatus* habitat *M. guttatus* (GG), *M. laciniatus* habitat *M. laciniatus* (LL), F_1_, F_2_, BCL, and BCG. Plants were stratified at UC Merced using the same methodology as the 2021 transplant (see above). We planted 75 blocks of 16 individuals (2 per genotype) at each site (hybrid site May 7th; *M. guttatus* site June 7th; *M. laciniatus* site July 13th). Due to limitations with germination, the total number of individuals at each site varied (Supplementary Table S1; Hybrid: 1,102 total, *M. guttatus*: 990 total, *M. laciniatus*: 1,112 total). Due to logistical constraints, we only measured the flowering time phenotype in our 2023 transplant. All other transplant methods were identical to the 2021 transplant (see above).

### Genotypic selection statistical methods

We identified fitness trade-offs between hybrid and parental genotypes at each site across years using linear mixed effects models in *nlme* (Pinheiro et al., 2023). We tested for genotype by environment interactions using each standardized phenotype as the dependent variable, site (E), genotype (G), and their interaction (G × E) as independent variables, and position nested in block as a random effect. All traits for models were standardized to the mean of zero and standard deviation of one (Kingsolver et al., 2001). We tested for differences in genotype fitness (survival and fecundity) by calculating mean fitness for every genotype and identified 95% confidence intervals using bootstrap estimates with 1,000 repetitions with replacement in the R package *boot* (Canty & Rippley, 2024; Davison & Hinkey, 1997).

### Phenotypic selection statistical methods

To examine the strength of selection (i.e., selection gradient *β*) on standardized traits, we used zero-truncated poisson and negative binomial linear mixed effect models that account for overdispersion of zeros in the seed set data in the R package *glmmTMB*, with position nested in block as a random effect (Brooks et al., 2017; Lande & Arnold, 1983; Mitchell-Olds & Shaw, 1987). We identified best fit models using *MuMin* (Bartoń, 2009). In 2023, models only include flowering time due to experimental constraints, so values reported are selection differentials, as they do not account for covariation among traits (Lande & Arnold, 1983). We ran phenotypic selection models in each site on (a) each genotype separately, (b) hybrid genotypes combined, and (c) all genotypes combined. To examine phenotypic correlations among traits in 2021, we ran a correlation analysis using the R package *corrplot* (Wei & Simko, 2021).

## Results

### Q1: hybrid environment fluctuates between being intermediate to more *M. laciniatus*-like

**Figure 3.**
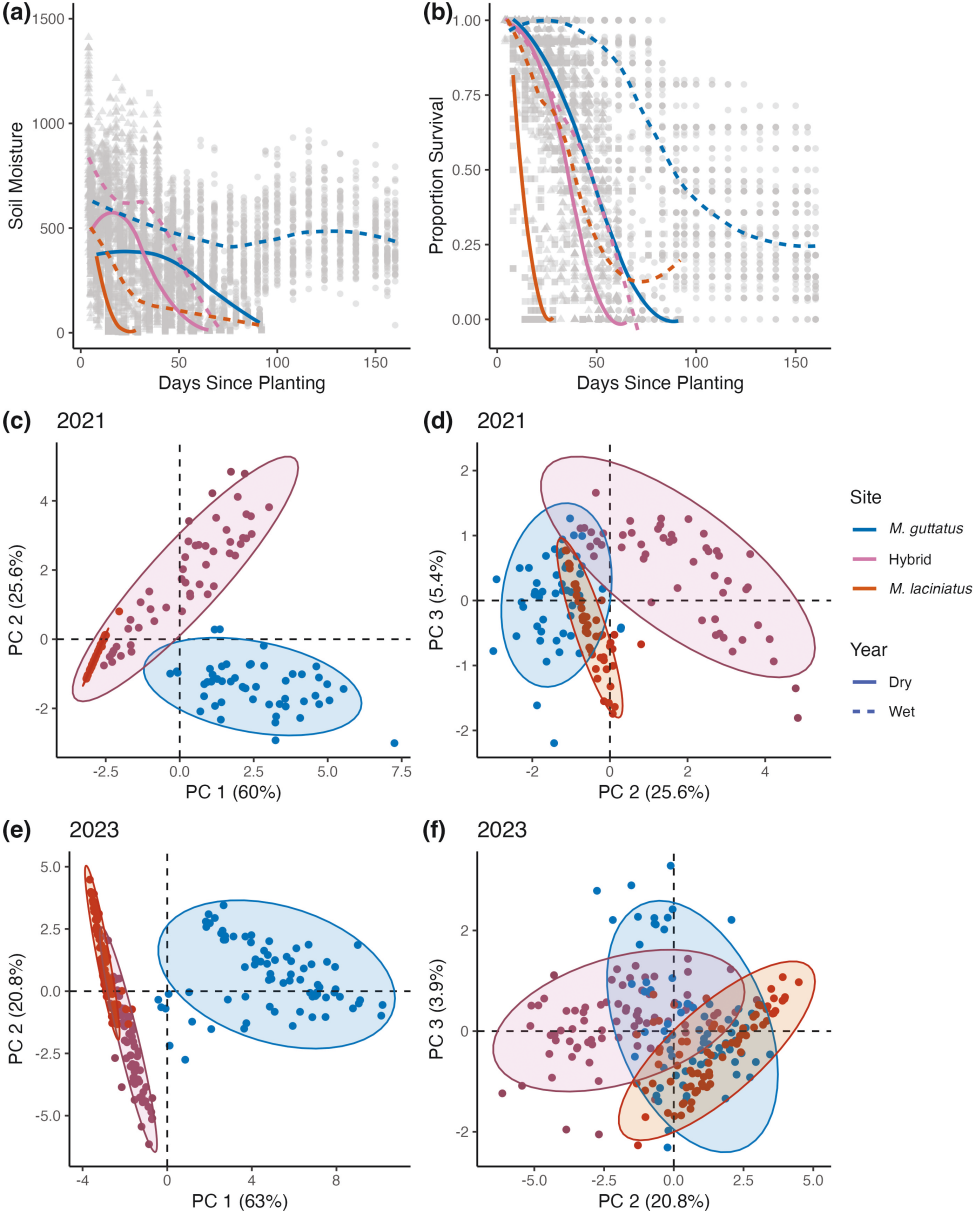
Soil moisture (A) and plant survival (B) decrease over time in *M. guttatus*, hybrid, and *M. laciniatus* sites. Solid lines connect weekly site means in 2021 and dotted lines connected weekly site means in 2023. Principal component analyses of soil moisture decrease over time in the dry year (C and D) and wet year (E and F). PC1 indicates differences in total soil moisture between sites (C and E), while PC2 and PC3 indicate differences in shape of soil moisture curves between sites (D and F).

To identify whether the hybrid site was intermediate to the two parental sites, we tested for significant differences in slope and shape of soil moisture decrease over time since planting (Figure 3). In both years the slope of hybrid site soil moisture decline was more similar to the *M. laciniatus* site (Pairwise comparisons 2021: *t*(1,587) = 5.227, *p *< 0.0001; 2023: *t*(2,732) = −5.368, *p *< 0.0001) than the *M. guttatus* site (Pairwise comparisons 2021: *t*(1,587) = 15.731, p < 0.0001; 2023: *t*(2,732) = 31.894, *p *< 0.0001). ANOVAs of soil moisture curve PCAs showed significant differences between all sites in each year, with variation in total soil moisture (PC1) in the hybrid zone intermediate to parental sites in the dry year (Supplementary Table S2; Figure 3C), and more similar to the *M. laciniatus* site in the wet year (Supplementary Table S2; Figure 3E). Soil moisture curve shape (PC2) was more similar in parental sites than the hybrid site in both years (Supplementary Table S2; Figure 3D and F). Therefore, the hybrid zone seems more similar to *M. laciniatus’s* habitat in overall soil moisture levels but unique from parent species in patterns of seasonal soil moisture decrease.

Soil moisture was critical for plant survival and flowering in each habitat and year. In models with years separated, soil moisture, time, site, and their interactions all had significant effects on plant survival across years (Figure 3; *p* < 0.0001). When we combined environmental data across years, year also had a significant effect on plant survival (Table 1, *p* < 0.0001). Soil surface temperature and ambient light levels were not in the best fit models for plant survival in either year (Table 1). This indicates that soil moisture had a strong effect on plant survival and varied over space and time both within a growing season and over years. There was higher proportion survival to flowering for every genotype in the wetter year (Supplementary Figure S1).

**Table 1.** Best fit linear mixed effect models for plant survival with all genotypes combined.

Year	Model	*k*	Log(L)	AIC	delta(i)	*wi*
2021	survival~time*site*soil moisture	14	742.46	−1456.92	0	0.99
2023	survival~time*site*soil moisture	12	590.67	−1157.33	0	0.99
Both	survival~time*site*soil moisture*year	21	1235.93	−2429.87	0	0.64

Herbivory had a significant effect on survival (dry year *f* = 41.72, *p* < 0.0001; wet year *f *= 14.2 *p *= 0.000167) but not fecundity in both years. Proportion herbivory varied significantly by site, year, genotype, and the interaction of all factors (Supplementary Table S3). The wet year had higher herbivory than the dry year in all habitats, while the hybrid and *M. guttatus* habitats had higher levels of herbivory than *M. laciniatus* in both years (Table 2). Unlike soil moisture, the hybrid zone is more like the *M. guttatus* habitat in herbivory levels (Table 2).

**Table 2.** Proportion of herbivory across all genotypes.

Site	Year	*N*	Proportion herbivory	*sd*	*se*	*ci*
*M. guttatus*	2021	3,404	0.148	0.356	0.006	0.012
*M. guttatus*	2023	974	0.173	0.378	0.012	0.024
Hybrid	2021	3,511	0.169	0.375	0.006	0.012
Hybrid	2023	1,030	0.285	0.452	0.014	0.028
*M. laciniatus*	2021	2,463	0.001	0.029	0.001	0.001
*M. laciniatus*	2023	1,112	0.043	0.203	0.006	0.012

### Q2: a lack of selection against hybrids in the hybrid zone

**Figure 4.**
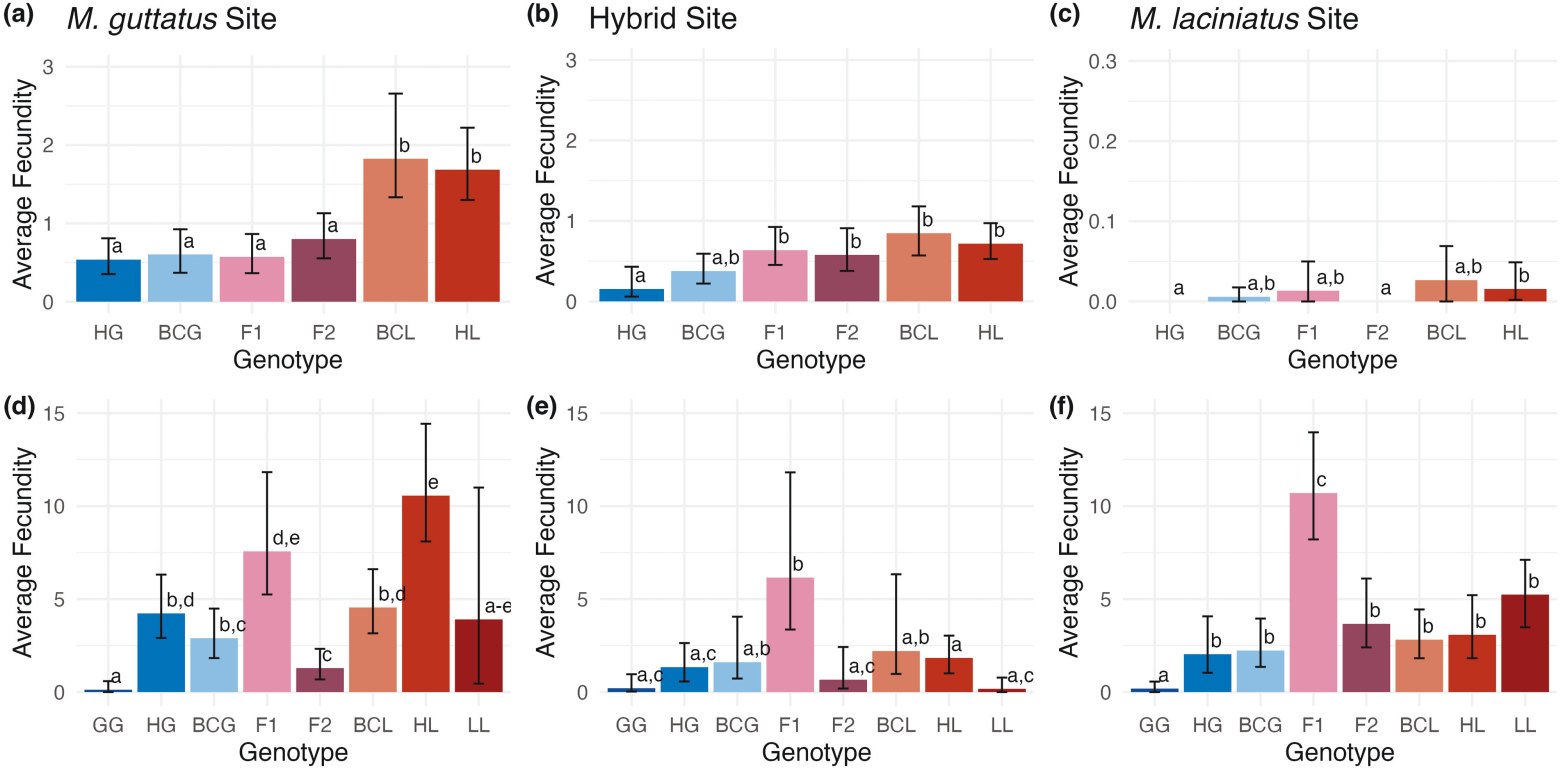
Average lifetime fecundity (total seed number/number of plants planted) per genotype in each site, with sites plotted separately. Upper row (A–C) is 2021 sites and lower row (D–F) is 2023 sites. Genotypes are, from left to right, *M. guttatus* habitat *M. guttatus* (GG), hybrid habitat *M. guttatus* (HG), back-crossed *M. guttatus* (BCG), first-generation hybrid (F_1_), second generation hybrid (F_2_), backcrossed *M. laciniatus* (BCL), hybrid habitat *M. laciniatus* (HL) and *M. laciniatus* habitat *M. laciniatus* (LL). Error bars represent 95% confidence intervals from bootstrap analysis. Values for average fecundity and 95% CI can be found in Supplementary Table 1.

There were genotypic fitness differences between habitats in both transplant years. In both years, we found no significant difference in fecundity between hybrid and parental genotypes in the hybrid habitat, with the exception of higher F_1_ hybrid fecundity in the wet year (Figure 4B and E; Supplementary Tables S1). In both years non-local *M. laciniatus* (HL) and BCL had higher fecundity (Figure 4A and D) and higher survival (Supplementary Figure S1) than most genotypes in *M. guttatus’* habitat. *M. guttatus* genotypes had the lowest total fecundity across all habitats, with pure-species habitat *M. guttatus* (GG) having even lower fecundity than hybrid zone *M. guttatus* (HG) in 2023 (Figure 4D). However, in that wet year ~40% of GG individuals survived to the end of the growing season without flowering in the *M. guttatus* habitat (Supplementary Figure S2). These results indicate a lack of selection against hybridization in the hybrid zone and possible local maladaptation in regard to fecundity in the *M. guttatus* habitat.

### Q3: genotypic selection varies temporally

We found temporal variation in selection between our dry and wet transplant years. There was higher fitness across genotypes and sites in the wet year compared to the dry year (Figure 4; Supplementary Figure S1). Patterns of which genotypes performed better also vary between years. In the dry year, genotypes with more *M. laciniatus* genetic background (HL, BCL) had higher fecundity and survival across sites, and most strongly in the *M. guttatus* site (Figure 4; Supplementary Figure S1).

During the wet year, we saw evidence of a hybrid advantage which was not present in the drier year (Figure 4). F_1_ hybrids had the highest fecundity in both the hybrid zone and *M. laciniatus* habitats, and second highest average fecundity in *M. guttatus’s* habitat (Figure 4D–F). F_2_ hybrids had significantly lower fitness than F_1_ hybrids in each habitat, where-as in the dry year they had similar levels of fitness in hybrid and *M. guttatus* habitats (Figure 4). The significant decrease in relative F_2_ fitness in the wetter year is likely due to expression of Bateson-Dobzhansky-Mueller incompatibilities, or BDMIs (Bateson, 1909; Dobzhansky, 1936; Muller, 1942).

### Q4: phenotypic selection for *M. laciniatus*-like traits in the hybrid zone

Genotype and environment effected quantitative trait expression in both transplant years. In the dry year, we found significant effects of site (E), plant genotype (G), and the interaction of site and genotype (G × E) on flowering time, plant height, stigma-anther separation, and leaf size (Supplementary Table S4). Site and genotype, but not their interaction, had significant effects on flower size and leaf lobing. In the wet year, site, genotype, and their interaction had a significant effect on flowering time (Supplementary Table S4).

**Figure 5.**
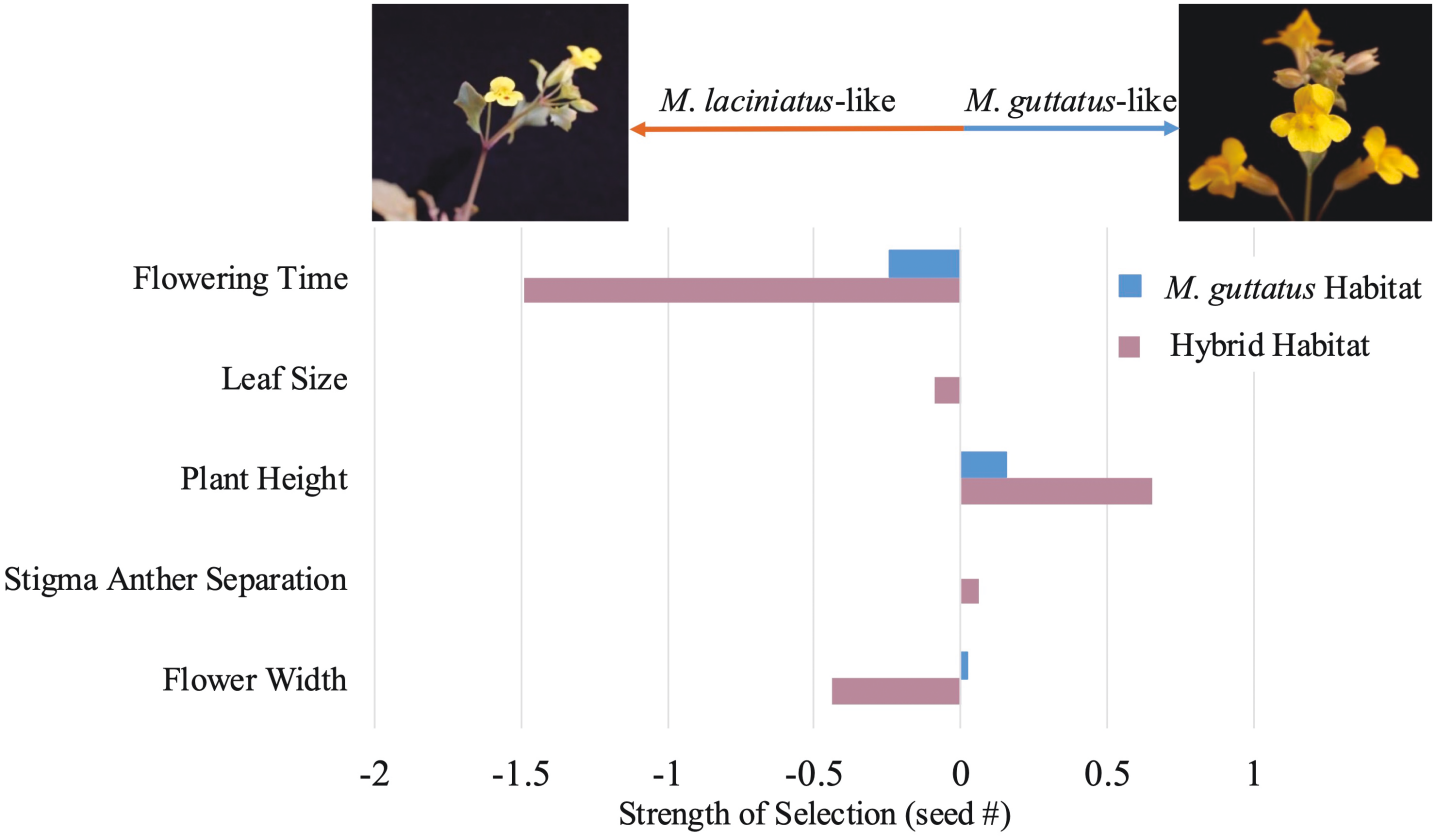
Phenotypic selection analysis in 2021 of all genotypes combined in the *M. guttatus* and Hybrid sites. There was no significant selection for leaf roundedness for genotypes combined in either species habitat in 2021. Pictures and arrows above the graph indicate which species’ traits the direction of selection is moving towards, *M. laciniatus* (left) and *M. guttatus* (right). Strength of selection refers to the selection gradient (*β*) from the zero-truncated poisson best fit model for all genotypes combined. Values from this graph can be found in Table 3, and model parameters can be found in Supplementary Table S6.

**Figure 6.**
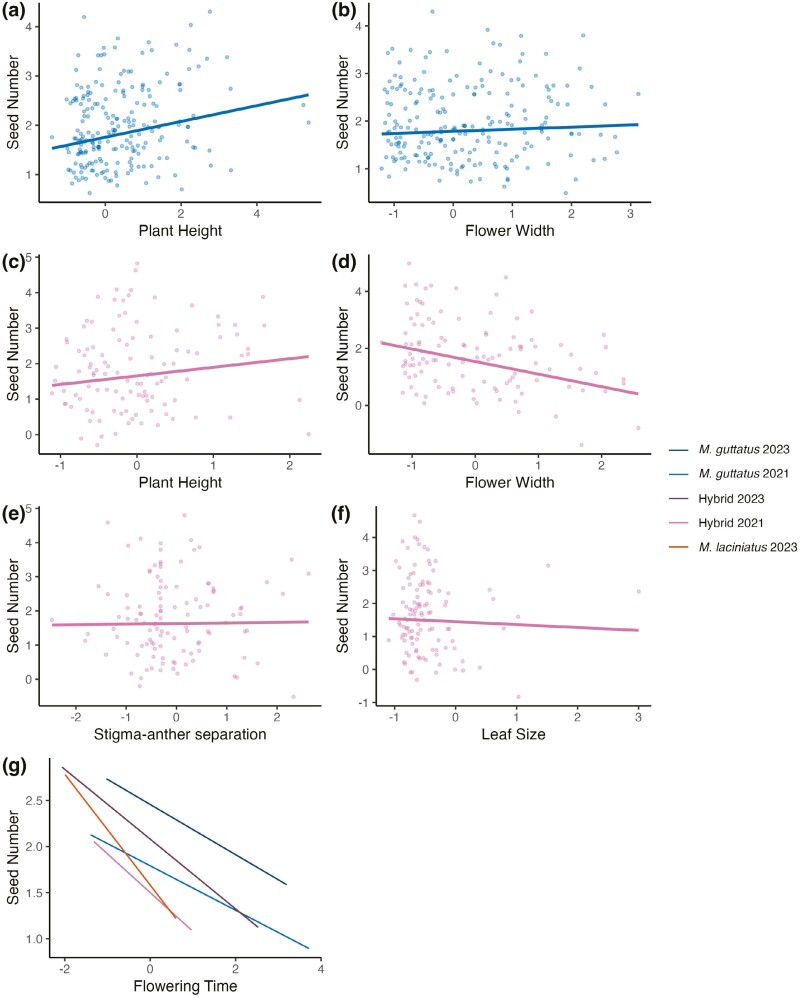
Directional selection on traits with all genotypes combined in *M. guttatus*, Hybrid, and *M. laciniatus* sites, visualized using partial residuals from multiple regression of best fit zero-truncated poisson models (Breheny & Burchett, 2017). The fitted curves show best fitting linear regression in 2021 (A–F) and both years (G). Plant height (A and C) and flower width (B and D) were in the best fit models for both sites, while stigma-anther separation (E) and leaf size (F) were only in best fit models for the hybrid habitat. Graph (G) also includes selection differentials for flowering time measured in 2023 in *M. guttatus*, hybrid and *M. laciniatus* habitats.

We conducted phenotypic selection analysis on plants in the *M. guttatus* and hybrid sites in 2021 and on flowering time across sites in 2023. Too few plants survived to flowering to perform selection analyses in the *M. laciniatus* site in 2021. Correlations among traits were relatively weak (*r* < 0.2, Supplementary Figure S3) except for size related phenotypes (*ρ* ~ 0.5). In our combined genotype zero-truncated poisson model we found stronger selection gradients in the hybrid zone than the *M. guttatus* site on most quantitative traits (Figure 5; Table 3; Supplementary Table S6). In the hybrid site, we found strong selection gradients for earlier flowering, smaller leaves, taller plants, and smaller flowers (Figure 6). These trait values are in the direction of *M. laciniatus* phenotypes, apart from taller plants, which is more *M. guttatus*-like. In the *M. guttatus* site we found weak selection gradients for earlier flowering and larger plants (Figures 4 and 6). In the hybrid site, the best fit zero-truncated poisson model involved interactions between flowering time, and plant height, leaf size, and stigma-anther separation, as well as an interaction between plant height and stigma-anther separation (Table 3). In contrast, there was only one interaction between flower width and plant height in the *M. guttatus* site (Table 3). These interactions indicate possible correlational selection (Svensson, 2023), suggesting stronger correlational selection in the hybrid habitat.

**Table 3.** Selection gradients (*β*) from the best fit zero-truncated poisson models based on 2021 seed number in the hybrid habitat (A) and M. guttatus habitat (B).

	HG	HL	BCL	BCG	F_1_	F_2_	All Hybrids	All Geno
(A)Hybrid Habitat								
Flower Width (FW)	–	–	0.733	0.588	–	−0.888^***^	−0.459^***^	−0.440^***^
Stigma Anther Separation (SA)	–	–	–	–	–	0.410	–	0.065
Plant Height (PH)	–	–	–	1.343^***^	0.431	–	0.699^***^	0.652^*^
Leaf Area (LA)	–	1.535	–	0.434^***^	–	–	0.022	−0.088
Flowering Time (FT)	–	–	0.726	−1.936^***^	−0.812	–	−1.474^***^	−1.490^***^
Leaf Roundedness (LR)	2.071	–	–	–	–	–	–	–
FT × FW	–	–	1.782^*^	–	–	–	–	–
FT × PH	–	–	–	–	–	–	1.109^**^	1.091
FT × LA	–	–	–	–	–	–	−1.879^**^	−2.058
FW × PH	–	–	–	−0.790^**^	–	–	–	–
FT × SA	–	–	–	–	–	–	–	0.044
SA × PH	–	–	–	–	–	–	–	0.047

(B)*M. guttatus* Habitat								
Flower Width (FW)	−0.364	−2.518^*^	0.629^**^	0.521^*^	–	0.191	–	0.022
Stigma Anther Separation (SA)	−1.671^**^	−1.640^**^	−0.447^*^	–	–	–	–	–
Plant Height (PH)	0.788	−4.537^*^	0.522^*^	–	0.404	–	0.185	0.157^*^
Leaf Area (LA)	−0.199^*^	−0.945^***^	0.426^*^	0.537^**^	0.376	–	0.13	–
Flowering Time (FT)	0.583	–	−0.415	–	−0.341^*^	–	−0.296^***^	−0.241^**^
Leaf Roundedness (LR)	0.54^***^	–	0.568^*^	0.693^**^	–	–	–	–
PH × SA	–	−3.524^*^	–	–	–	–	–	–
PH × FT	−0.986^*^	–	–	–	0.275^*^	–	0.126	–
PH × FW	−0.503	−5.354^*^	–	–	–	–	–	0.045^*^
LR × SA	−1.28^*^	–	−0.858^*^	–	–	–	–	–
FT × FW	−0.689	–	–	–	–	–	–	–
LA × LR	−0.264^*^	–	−0.064	–	–	–	–	–
LA × SA	1.419^***^	–	1.085^**^	–	–	–	–	–
PH × LA	–	–	–	–	–	–	−0.035	–
FW × SA	–	−1.2157	–	–	–	–	–	–

*β* indicates strength and direction of selection, while asterisks indicate significance:

^*^ *p* < 0.05.

^**^ *p* < 0.01.

^***^ *p* < 0.001.

Model parameters for this data can be found in Supplementary Table S6.

When we broke up the phenotypic selection analysis by genotype, we found that in both zero-truncated poisson and negative binomial models, the hybrid genotypes (F_1_, F_2_, BCL, BCG) in the *M. guttatus* site largely experienced selection towards *M. guttatus* trait values (Supplementary Figures S4 and S5; Table 3, Supplementary Tables S5 and S6). In the negative binomial, most genotypes in the hybrid habitat experienced selection towards *M. laciniatus*-like traits (earlier flowering, smaller leaves, smaller plants, and smaller flowers) except for *M. guttatus* (HG) which experienced selection for more *M. guttatus*-like traits: later flowering, larger flowers, and greater stigma-anther distance (Supplementary Figure S5; Supplementary Tables S5 and S6). In the wet year, we found selection differentials in the direction of earlier flowering in all sites and models, with the strongest selection for early flowering in the *M. laciniatus* and hybrid sites (Figure 6G, Table 4 and Supplementary Table S7). Therefore, in both years, we found selection in the hybrid zone for more *M. laciniatus*-like traits.

**Table 4.** 2023 selection differentials (*s*) on flowering time from the best fit (A) zero-truncated poisson and (B) negative binomial models based on seed number.

Site	GG	HG	BCG	F_1_	F_2_	BCL	HL	LL	All Hybrids	All Geno
	(A) Truncated Poisson Selection on Flowering Time									
*M. guttatus*	–	−0.203	−0.668^*^	−0.412	0.170	−0.599	−0.527^*^	0.104	−0.210	−0.272^*^
Hybrid	–	−0.247	−1.912^*^	−0.733	0.724	−0.391	−0.548	–	−0.659^*^	−0.378^*^
*M. laciniatus*	–	−0.315	−0.256	−0.951^***^	−0.715	−1.443^**^	−0.304	0.243	−0.735^***^	−0.602^***^

	(B) Negative Binomial Selection on Flowering Time									
*M. guttatus*	–	−0.091	−0.123	0.039	−0.111	−0.28	0.111	–	−0.035	−0.013
Hybrid	–	−0.241	−1.025	−0.152	0.312	−0.413	0.097	–	−0.243	−0.153
*M. laciniatus*	–	−0.349	−0.440	−0.285	0.165	−0.285	−0.145	−0.305	−0.254	−0.256^*^

*s* indicates strength and direction of selection on one trait, while asterisks indicate significance:

^*^ *p* < 0.05.

^**^ *p* < 0.01.

^***^ *p* < 0.001.

Model parameters for these analyses can be found in Supplementary Table S7.

## Discussion

The contribution of hybridization to adaptation and speciation has long interested evolutionary biologists. To investigate the evolutionary forces maintaining hybrid zones between two sympatric Monkeyflower species, we performed repeated reciprocal transplants between native hybrid and pure species’ habitats. We predicted that hybridization is maintained in *M. guttatus-M. laciniatus* hybrid zones by a hybrid fitness advantage, while parental species’ habitats have decreased hybrid fitness and increased fitness of local genotypes (Figure 2). Depending on the year, we either found selection for, or a lack of selection against, hybrids in the hybrid zone and local maladaptation in the *M. guttatus* habitat. There was evidence of temporally varying positive and negative selection acting on hybrids, with selection for *M. laciniatus*-like traits in the hybrid zone in the dry year and fitness advantage of F_1_ hybrids but expression of genetic incompatibilities in F_2_ hybrids in the wet year. Identifying how hybridization interacts with natural selection is essential for understanding how plant populations will persist in future changing conditions.

### A shifting hybrid zone

Hybridization might be adaptive in the wild if the hybrid zone is environmentally intermediate between parental species’ habitats. To identify whether our hybrid zone was environmentally intermediate between *M. laciniatus* and *M. guttatus’s* habitats, we compared fine-scale environmental variation and its effect on plant fitness between sites. Soil moisture decrease was strongly associated with plant survival in our experiments, confirming findings of previous studies in this system (Ferris &Willis, 2018; Tataru et al., 2023). The hybrid site had soil moisture levels and curves that fluctuated between years and were either intermediate to parental sites (dry year), or more similar to *M. laciniatus’* rocky outcrop habitat (wet year). Temporal variation in differentiation between hybrid and parental species’ environments and equal fitness of hybrids and parental species in hybrid zones has also been found in Louisiana *Irises* (Emms & Arnold, 1997). While hybrid zones as clines between parental species’ habitats have been well described (reviewed in Arnold, 1997), not many studies have investigated how temporal variation in environmental variables effect the maintenance of hybridization. Although soil moisture was sometimes more similar to *M. laciniatus’s* rocky habitat, high herbivory levels in the hybrid zone in both years were more similar to *M. guttatus’s* meadow habitat and the combination of these divergent environmental pressures could cause a hybrid’s unique blend of parental alleles to be adaptive. Examining the influence of multiple kinds of environmental variation constructs a more holistic picture of whether a hybrid zone is truly ecologically intermediate or unique compared to parental habitats.

### Lack of selection against hybrids in a hybrid zone

Given that our hybrid habitat was environmentally intermediate in some years, we next sought to identify whether hybrids had a fitness advantage in the hybrid habitat. Instead of clear selection for hybridization, we found that hybrids had similar fitness to parentals in the hybrid habitat in both years, with the exception of high F_1_ fitness in the wet year. Surprisingly, we did not find an advantage of local genotypes in each parental habitat. Instead, *M. laciniatus* had higher total fitness than *M. guttatus* in *M. guttatus’s* habitat in both years (Figure 4). In the extremely high snowpack year, non-local *M. laciniatus* had significantly higher fitness in the *M. guttatus* meadow habitat than in its own rocky outcrop environment. This asymmetry in parental fitness suggests that *M. laciniatus* is better adapted to both species habitats and could indicate a shifting fitness landscape in *M. guttatus’s* meadows due to recent climate change.

Lack of selection against hybrids may be due to the environmentally intermediate nature of our hybrid zone (Figure 3). A mixture of drought-adapted *M. laciniatus* alleles with more mesic *M. guttatus* genetic background could have similar fitness to parental species over time in a hybrid environment with soil moisture levels that fluctuate between the two species’ habitats. In dry years like 2021, it is potentially adaptive to have a higher proportion of drought-adapted-*M. laciniatus* genetic background as seen by BCL hybrids having the highest fitness and BCG having higher fitness than pure *M. guttatus* in the hybrid zone (Figure 4B). This pattern of *M. laciniatus* alleles being advantageous in an *M. guttatus* background would be consistent with patterns of asymmetric introgression in other self-fertilizing-outcrossing species pairs. Genomic analyses of gene flow between *Mimulus nasutus* and *M. guttatus* found asymmetric and recurring introgression of the drought-adapted self-fertilizing species into *M. guttatus* (Kenney & Sweigart, 2016). This same pattern was detected between *Clarkia xantiana* (self-fertilizing) and *Clarkia parviflora* (outcrossing), as well as evidence of increased introgression between species when spring precipitation is more variable (Sianta et al., 2024).

### Fluctuating selection influences hybrid fitness

We found that the strength of selection fluctuates temporally in a *M. laciniatus*-*M. guttatus* hybrid zone. The wetter year transplant had higher overall fitness than the drier year, and fitness differences between genotypes varied temporally, with F_1_ hybrids having an advantage in the wetter year. Our findings of an environmentally dependent hybrid advantage (Figure 4) parallel other findings of strong G × E interactions in a greenhouse common garden with differing watering regimes of Louisiana *Iris* species and hybrids (Johnston et al., 2001). Also in the wet year, a larger proportion of local *M. guttatus* survived to the end of the season in the *M. guttatus* habitat but never flowered, suggesting a shift in life history expression. In populations of *Streptanthus tortuousus* with variable life history expression, plants with later germination timing are more likely to perennate (Gremer et al., 2020). While we planted cotyledons (not seeds) due to logistical constraints, we planted our seedlings later in the high snowpack year, which could have led to a proportion of the *M. guttatus*, which are known to be facultatively perennial at high elevations (Friedman & Willis, 2013), behaving as perennials. Our findings demonstrate how annual environmental variation plays an important role in life history cues and subsequent population structure.

Another observed fluctuation in genotypic selection was that in the wetter year, we found low F_2_ relative to F_1_ fitness in each habitat, but similar fitness between the two hybrid generations in the dry year (Figure 4). This pattern of low F_2_ relative fitness is consistent with the expression of genetic incompatibilities between the two species that are masked in the heterozygous F_1_ but expressed in later generation hybrids (Coyne & Orr, 2004). Previous studies of greenhouse crosses between *M. guttatus* and *M. laciniatus* have not found hybrid breakdown (Vickery, 1964; Ferris et al., 2017), further suggesting that the expression of these BDMIs may be environmentally dependent. Environmental dependence, or extrinsic post-zygotic reproductive isolation, is consistent with the fitness difference only appearing in the wet year. A field transplant with multiple ecotypes and hybrids of *Senecio lautus* also found an environmentally dependent decrease in F_2_ relative to F_1_ fitness (Walter et al., 2020). The environmentally dependent expression of BDMIs in later generation hybrids might balance any selective advantage experienced by early generation hybrids or drought-adapted-*M. laciniatus* alleles in the hybrid zone. Experimental hybrids used in the wetter year were created from a subset of genotypes used in the dry year, but the strength of the temporal fluctuations in fitness across genotypic categories makes it unlikely that these patterns are driven by differences in genetic variation alone.

### Drought-adapted traits are advantageous in the hybrid zone

*Mimulus laciniatus* is adapted to harsh and ephemeral habitats, so genetic material from this species may help with adaptation to dry conditions like those seen in 2021 and in the hybrid zone (Figure 3). The direction of phenotypic selection in the hybrid zone was largely towards *M. laciniatus*-like trait values, with the strongest selection on traits involved in reproductive isolation: flowering time and flower size. Adaptive introgression in hybrids has been demonstrated in a number of systems (Seehausen, 2004), such as in *Helianthus* where there has been adaptative introgression of abiotic tolerance (Whitney et al., 2010) and herbivory resistance (Whitney et al., 2006). Subsequent experimental Sunflower hybrid transplants found increased hybrid fitness and faster trait evolution in hybrid than non-hybrid populations (Mitchell et al., 2019). This suggests that patterns of selection for *M. laciniatus*-like traits in the hybrid zone are a signature of adaptive introgression.

### Conclusions

Our findings suggest that in this system hybridization is maintained by a lack of selection against hybridization in the hybrid zone and temporally varying selection (Arnold, 1997). Gene flow between our incompletely reproductively isolated species may allow for advantageous introgression of drought-adapted traits from a rocky outcrop specialist, *M. laciniatus,* into the more mesic-adapted *M. guttatus* in drier years. This is possibly due to the hybrid zone being environmentally intermediate in some ways. Novel combinations of traits in hybrids paired with strong G × E interactions may provide a fitness benefit; however, BDMIs expressed in later generation hybrids could limit this benefit in years with high water availability, a form of migration-selection balance (Key, 1968). While selection varied temporally in one hybrid zone, identifying whether selection varies across multiple *Mimulus* hybrid zones would broaden understanding of the maintenance of hybridization and how patterns of gene flow vary across space as well as time. Overall, our study demonstrates that large-scale reciprocal transplants are important in identifying how variation in natural selection impacts the maintenance of natural hybrid zones.

## Supplementary material

Supplementary material is available online at *Evolution Letters*.

qrae050_suppl_Supplementary_Tables_S1-S7_Figures_S1-S5

## Data Availability

Data and code available from the Dryad Digital Repository (https://doi.org/10.5061/dryad.k98sf7mg4).
