# Supplementary material for: Fluctuating selection in a monkeyflower hybrid zone
Source: Evol Lett. 2024 Sep 26;9(1):77–88. doi: 10.1093/evlett/qrae050 (PMC11790225; doi:10.1093/evlett/qrae050)
Supplement: qrae050_suppl_Supplementary_Tables_S1-S7_Figures_S1-S5 [file qrae050_suppl_supplementary_tables_s1-s7_figures_s1-s5.pdf]

Supplementary Figures

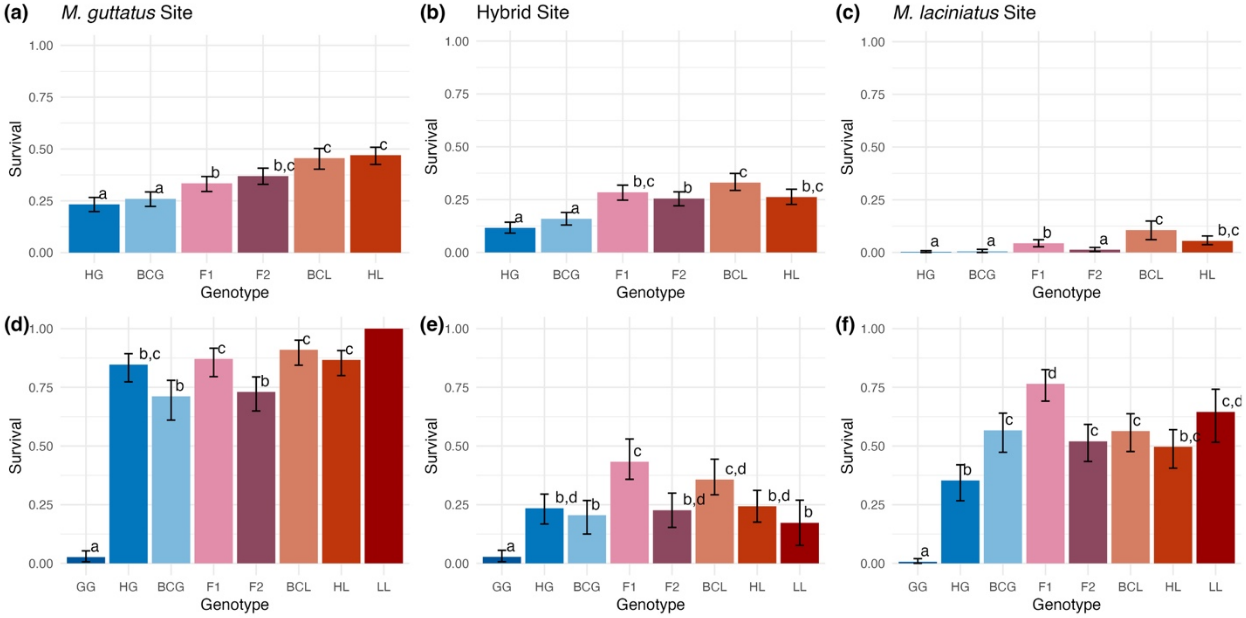

S1. Average proportion of plants that survived to flowering per genotype in each site, with sites plotted separately. Upper row (a, b, c) is 2021 sites and lower row (d, e, f) is 2023 sites. Genotypes are, from left to right, *M. guttatus* habitat *M. guttatus* (GG), hybrid habitat *M. guttatus* (HG), back-crossed *M. guttatus* (BCG), first generation hybrid (F_1), second generation hybrid (F_2), backcrossed *M. laciniatus* (BCL), hybrid habitat *M. laciniatus* (HL) and l *M. laciniatus* habitat *M. laciniatus* (LL). Error bars are calculated using 95% confidence intervals from bootstrap analysis.

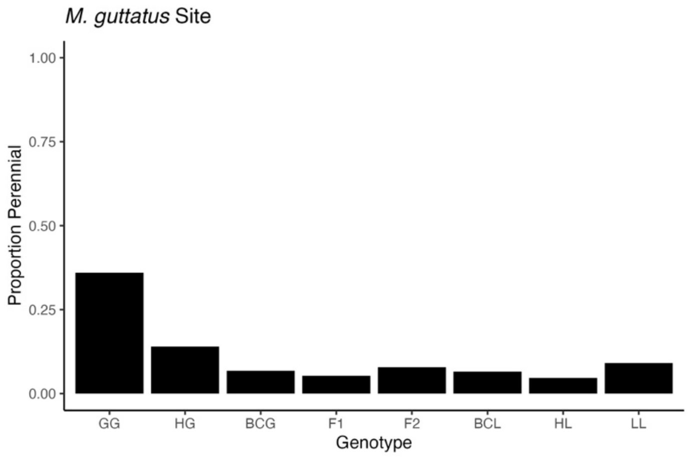

S2. Proportion of individuals planted in the *M. guttatus* site in 2023 that survived until the end of the season without flowering (proxy for perenniality).

	Flowering Tin	Leaf Lobing	Flower Width	Plant Height	Stigma Anther Sep.	Leaf Area
Flowering Time	1.00					
Leaf Lobing	0.16	1.00				
Flower Width	0.15	-0.10	1.00			
Plant Height	0.24	-0.07	0.58	1.00		
Stigma Anther Sep.	0.15	-0.04	0.36	0.26	1.00	
Leaf Area	-0.20	-0.15	0.42	0.41	0.20	1.00

S3. Trait correlations of standardized traits for all genotypes and sites in 2021. Correlations did not vary significantly between sites.

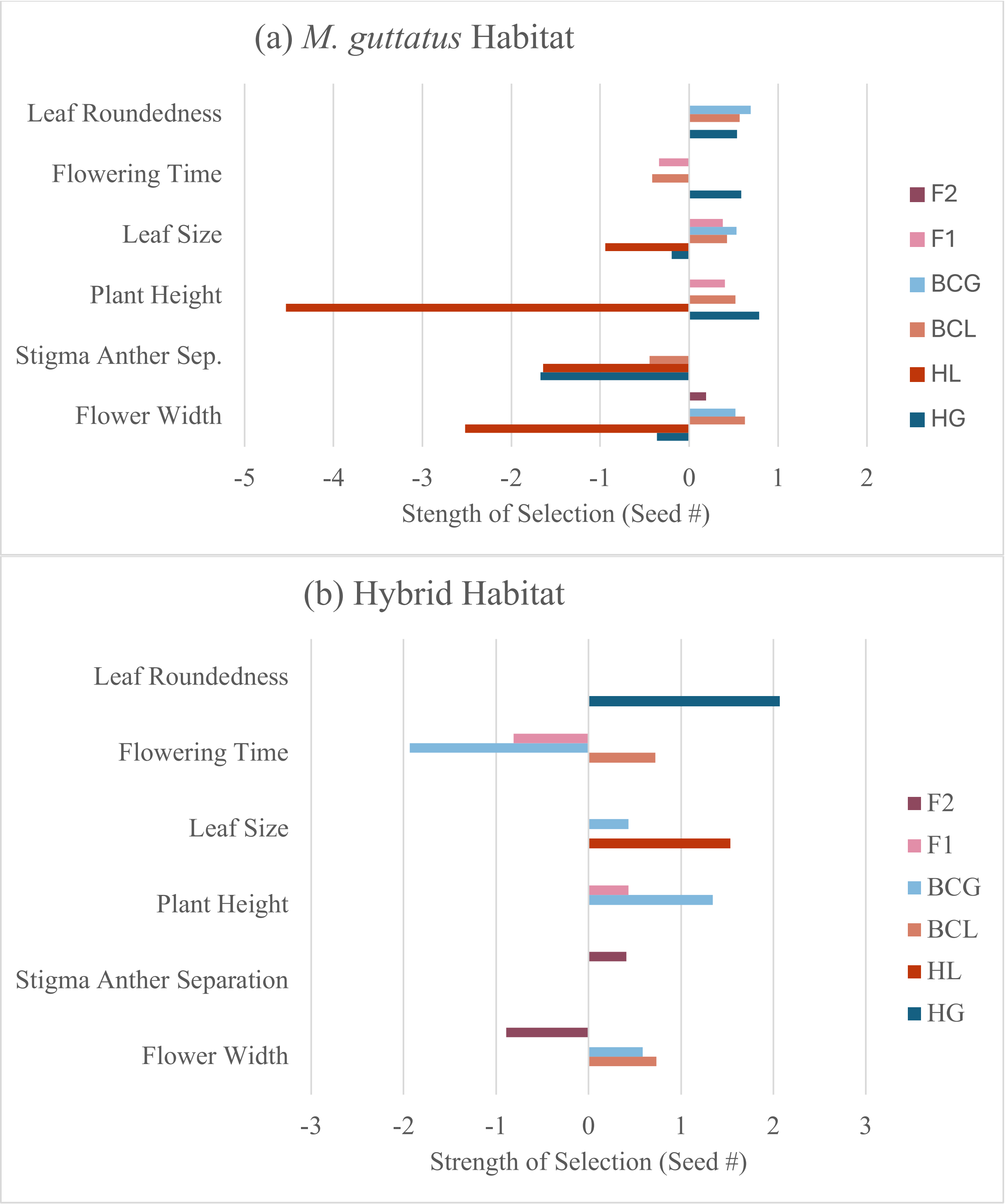

S4. Phenotypic selection gradients from zero-truncated poisson models of the 2021 transplant in the *M. guttatus* (a) and hybrid (b) sites. Values from these graphs can be found in Table 3, and model parameters can be found in supplementary table S6.

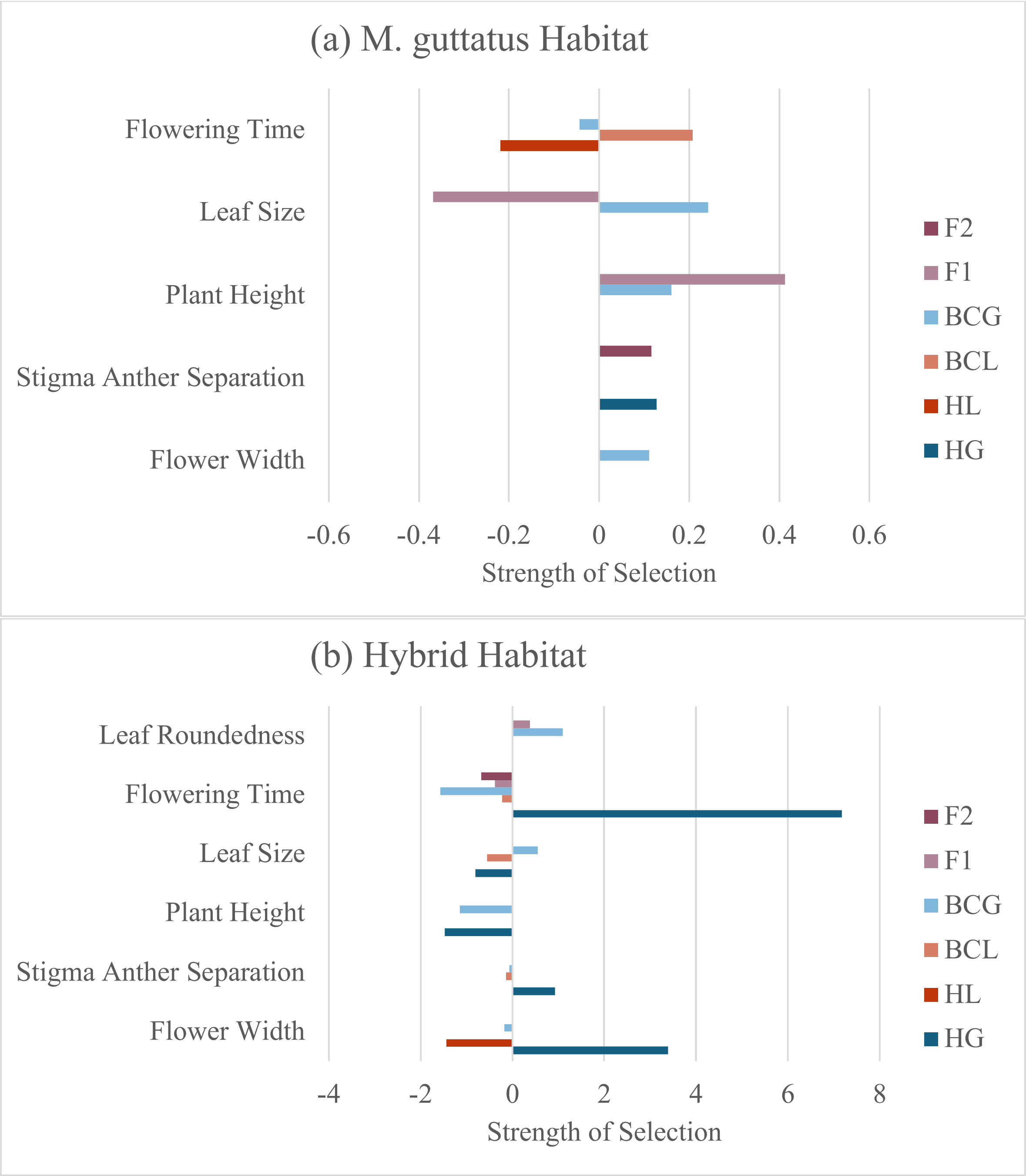

S5. Phenotypic selection gradients from negative binomial of the 2021 transplant in the *M. guttatus* (a) and hybrid (b) sites. Values from these graphs can be found in supplementary table S4, and model parameters can be found in supplementary table S6.

Supplementary Tables

Year	Site	Genotype	# Planted	Date Planted	Avg. Fecundity	95% CI
2021	*M. guttatus*	HG	600	4/28/21	0.537	0.3527, 0.81
2021	*M. guttatus*	BCG	600	4/28/21	0.604	0.369, 0.925
2021	*M. guttatus*	F1	600	4/28/21	0.573	0.364, 0.864
2021	*M. guttatus*	F2	600	4/28/21	0.8	0.554, 1.13
2021	*M. guttatus*	BCL	372	4/28/21	1.826	1.333, 2.657
2021	*M. guttatus*	HL	600	4/28/21	1.685	1.299, 2.221
2021	Hybrid	HG	600	4/9/21	0.155	0.06, 0.431
2021	Hybrid	BCG	600	4/9/21	0.376	0.221, 0.592
2021	Hybrid	F1	600	4/9/21	0.636	0.453, 0.925
2021	Hybrid	F2	600	4/9/21	0.578	0.379, 0.909
2021	Hybrid	BCL	498	4/9/21	0.847	0.571, 1.181
2021	Hybrid	HL	600	4/9/21	0.718	0.527, 0.972
2021	*M. laciniatus*	HG	300	5/26/21	0	NA
2021	*M. laciniatus*	BCG	342	5/26/21	0.006	0, 0.018
2021	*M. laciniatus*	F1	600	5/26/21	0.013	0, 0.05
2021	*M. laciniatus*	F2	462	5/26/21	0	NA
2021	*M. laciniatus*	BCL	144	5/26/21	0.027	0, 0.069
2021	*M. laciniatus*	HL	510	5/26/21	0.016	0.002, 0.049
2023	*M. guttatus*	GG	150	6/7/23	0.127	0, 0.588
2023	*M. guttatus*	HG	150	6/7/23	4.233	2.909, 6.318
2023	*M. guttatus*	BCG	118	6/7/23	2.9	1.828, 4.489
2023	*M. guttatus*	F1	138	6/7/23	7.568	5.249, 11.828
2023	*M. guttatus*	F2	150	6/7/23	1.291	0.681, 2.326
2023	*M. guttatus*	BCL	122	6/7/23	4.549	3.160, 6.608
2023	*M. guttatus*	HL	150	6/7/23	10.567	8.10, 14.43
2023	*M. guttatus*	LL	12	6/7/23	3.909	0.455, 11
2023	Hybrid	GG	150	5/7/23	0.208	0.028, 0.958
2023	Hybrid	HG	150	5/7/23	1.342	0.570, 2.636
2023	Hybrid	BCG	150	5/7/23	1.607	0.727, 4.054
2023	Hybrid	F1	150	5/7/23	6.157	3.363,11.815
2023	Hybrid	F2	150	5/7/23	0.664	0.190, 2.423
2023	Hybrid	BCL	150	5/7/23	2.208	0.974, 6.335
2023	Hybrid	HL	150	5/7/23	1.838	1.007, 3.036
2023	Hybrid	LL	52	5/7/23	0.173	0, 0.78
2023	*M. laciniatus*	GG	150	7/13/23	0.188	0, 0.5638
2023	*M. laciniatus*	HG	150	7/13/23	2.04	1.040, 4.072
2023	*M. laciniatus*	BCG	150	7/13/23	2.233	1.353, 3.952
2023	*M. laciniatus*	F1	150	7/13/23	10.705	8.21, 13.97
2023	*M. laciniatus*	F2	150	7/13/23	3.671	2.404, 6.105
2023	*M. laciniatus*	BCL	150	7/13/23	2.819	1.819, 4.444
2023	*M. laciniatus*	HL	150	7/13/23	3.08	1.821, 5.212
2023	*M. laciniatus*	LL	62	7/13/23	5.242	3.484, 7.113

Table S1. Number of plants per genotype planted at each site in each year with date planted. Average fecundity (total seed number/number of plants planted) per genotype in each site with 95% confidence intervals from bootstrap estimates with 1000 repetitions with replacement.

PC	Year	Site		Block		Pairwise Site Difference		
		F	*p-value*	*F*	*p-value*	H-G	L-G	L-H
1	2021	211	<2e-16	3.52	0.06	-2.33	-5.37	-3.04
1	2023	555.88	<2e-16	3.95	0.05	-6.71	-7.85	-1.14
2	2021	137.9	<2e-16	1.21	0.273	3.21	0.92	-2.29
2	2023	120.8	<2e-16	107.6	<2e-16	-2.52	0.82	3.34
3	2021	22.08	4.19e-09	2.94	0.09	0.43	-0.47	-1.22
3	2023	9.13	0.0002	1.16	0.28	NS	-0.57	-0.54

Table S2. Results of ANOVAs of soil moisture PCA analysis, with PC axis as dependent variable and site and block as independent variables. Pairwise site difference refers to significant differences in Tukey post hoc tests of ANOVAs.

Independent Variable	*df*	*F statistic*	*p value*
Site	2	336.262	< 2e-16
Genotype	7	6.143	3.43e-07
Year	1	98.206	< 2e-16
Site x Year	2	25.152	1.25e-11
Site x Genotype	14	3.426	1.37e-05
Genotype x Year	5	7.819	2.33e-07
Site x Genotype x Year	10	4.508	2.17e-06

Table S3. Results of best fit linear mixed effects models for effect of site, genotype, year, and their interaction (independent variables) on herbivory (dependent variable).

Trait	Genotype (G)		Environment (E)		G x E	
	F statistic	*p value*	*F statistic*	*p value*	*F statistic*	*p value*
Flowering Time	130.3	< 0.001	102.1	< 0.001	8.6	< 0.001
Plant Height	110.7	< 0.001	29.3	< 0.001	5.2	< 0.001
Stigma-Anther Sep.	60.4	< 0.001	11.3	< 0.001	2.6	0.007
Leaf Size	21.6	< 0.001	68.7	< 0.001	2.7	0.004
Flower Size	140.3	< 0.001	18.6	< 0.001	1.4	0.1938
Leaf Lobing	4.1	0.0015	65.5	< 0.001	0.9	0.5237
Flowering Time (2023)	22.1	< 0.001	618.6	< 0.001	6.2	< 0.001

Table S4. Results of linear mixed effects models of effect of genotype, environment, and their interaction (independent variables) on trait values (dependent variable) for all sites combined in 2021, and for flowering time in 2023.

(a) Hybrid Habitat

Trait	HG	HL	BCL	BCG	F1	F2	All Hybrids	All Geno
Flower Width (FW)	3.396	-1.439	-	-0.179	-	-	-	-
Stigma Anther Separation (SA)	0.927	-	-0.144	-0.066	-		-	-
Plant Height (PH)	-1.48	-	-	-1.144	-	-	-	-
Leaf Area (LA)	-0.805	-	-0.549	0.550	-	-	-	-
Flowering Time (FT)	7.171	-	-0.223	-1.568	-0.379	-0.676	-0.439	-0.427
Leaf Roundedness (LR)	-	-	-	1.090	0.379	-	-	-
FT x FW	-6.525	-	-	0.160	-	-	-	-
FT x SA	-	-	-0.055	-	-	-	-	-
FT x PH	1.476	-	-	-2.650	-	-	-	-
FW x PH	-	-	-	0.610	-	-	-	-

(b) *M. guttatus* habitat

Trait	HG	HL	BCL	BCG	F1	F2	All Hybrids	All Geno
Flower Width (FW)	-	-	-	0.111	-	-	-	-
Stigma Anther Separation (SA)	0.127	-	-	-	-	0.116	-	-
Plant Height (PH)	-	-	-	0.161	0.413	-	0.109	-
Leaf Area (LA)	-	-	-	0.242	-0.37	-	-	-
Flowering Time (FT)	-	-0.21	0.208	-0.044	-	-	-	-
Leaf Roundedness (LR)	-	-	-	-	-	-	-0.173	0.101
FT x PH	-	-	-	-0.052	-	-	-	-
FT x FW	-	-	-	-0.022	-	-	-	-

Table S5. Selection gradients (β) from the best fit negative binomial; models based on 2021 seed number in the hybrid habitat (A) and *M. guttatus* habitat (B). β indicates strength and direction of selection. Model parameters for this data can be found in supplementary table S6.

Site	Geno	Model	Best Fit Model	n	Standard Error	AIC	Log-L
Hybrid	HG	poisson	seed ~ LL	6	1.34	24.8	-8.42
Hybrid	HL	poisson	seed ~ LA	17	1.23	106.4	-49.2
Hybrid	BCL	poisson	seed ~ FT * FW	26	0.55/0.44	173.2	-80.6
Hybrid	BCG	poisson	seed ~ FT + FW * PH + LA	13	0.4/0.43/0.26/0.11	66.1	-25.05
Hybrid	F1	poisson	seed ~ FT + PH	27	0.52/0.25	150	-70
Hybrid	F2	poisson	seed ~ FW + SA	24	0.27/0.27	144.24	-67.12
Hybrid	hybrid	poisson	seed ~ FT * PH + FW + LA * FT	90	0.43/0.2/0.13/0.21	658	-320
Hybrid	all	poisson	seed ~ FT * SA * PH + FW + SA + LA * FT	113	0.43/0.14/0.22/0.13/0.2	657.9	-316
M. guttatus	HG	poisson	seed ~ FT * FW * PH * SA * LA * LL	24	0.53/0.29/0.65/0.58/0.08/0.16	131	-48.5
M. guttatus	HL	poisson	seed ~ FW * PH * SA +LA	46	1/1.95/0.54/0.20	281.1	-131.53
M. guttatus	BCL	poisson	seed ~ FT + FW +PH + SA * LA * LL	42	0.24/0.24/0.25/0.22/0.18/0.23	260.1	-117.06
M. guttatus	BCG	poisson	seed ~ FW + LA + LL	32	0.23/0.21/0.23	195.75	-91.88
M. guttatus	F1	poisson	seed ~ FT * PH + LA	33	0.16/0.21/0.22	177.85	-81.93
M. guttatus	F2	poisson	seed ~ FW	37	0.19	247.9	-120.43
M. guttatus	hybrid	poisson	seed~ FT * PH + LA * PH	144	0.09/0.110.09	1335.6	-659.8
M. guttatus	all	poisson	seed ~ FT + FW * PH	214	0.09/0.1/0.12	1334	-660
M. laciniatus	HG	poisson	sample size too small	0	-	-	-
M. laciniatus	HL	poisson	sample size too small	0	-	-	-
M. laciniatus	BCL	poisson	sample size too small	2	-	-	-
M. laciniatus	BCG	poisson	sample size too small	1	-	-	-
M. laciniatus	F1	poisson	sample size too small	0	-	-	-
M. laciniatus	F2	poisson	sample size too small	0	-	-	-
M. laciniatus	hybrid	poisson	sample size too small	0	-	-	-
M. laciniatus	all	poisson	sample size too small	3	-	-	-
Hybrid	HG	binomial	seed ~ FT * FW + FT * PH + SA + LA	20	6.02/2.23/1.54/0.79/1.22	40.1	-9
Hybrid	HL	binomial	seed ~ FW	27	1.34	58.32	-24.16
Hybrid	BCL	binomial	seed ~ FT * SA + LA	45	0.51/0.38/0.7	95	-39.8
Hybrid	BCG	binomial	seed ~ FW * PH + FT * PH + FT * FW + LA + LL + SA	31	0.64/0.88/1.8/0.45/0.38/0.8	66.2	-20.1
Hybrid	F1	binomial	seed ~ FT * FW * PH + SA + LA * LL	45	0.9/0.36/0.55/0.31/0.61/0.6	103.6	-36.8
Hybrid	F2	binomial	seed ~ FT	53	0.47	93.8	-41.89
Hybrid	hybrid	binomial	seed ~ FT	174	0.23	304.7	-147.3

Hybrid	all	binomial	seed ~ FT	221	0.2	382.5	-186.3
M. guttatus	HG	binomial	seed ~ SA	46	0.25	88.97	-39.485
M. guttatus	HL	binomial	seed ~ FT	74	0.3	145.16	-67.58
M. guttatus	BCL	binomial	seed ~ FT	67	0.18	132	-61.6
M. guttatus	BCG	binomial	seed ~ FW * FT + LA + PH * FT	66	0.26/0.24/0.17/ 0.31	-672.9	346.45
M. guttatus	F1	binomial	seed ~ PH + LA	73	0.2/0.26	125.1	-56.5
M. guttatus	F2	binomial	seed ~ SA	72	0.18	132.9	-61.4
M. guttatus	hybrid	binomial	seed ~ PH + LA	278	0.08/0.08	-	-
M. guttatus	all	binomial	seed ~ LL	398	0.08	702.1	-346.1
M. laciniatus	HG	binomial	sample size too small	1	-	-	-
M. laciniatus	HL	binomial	seed ~ FW * SA + LL	6	-	16	-1.35E-13
M. laciniatus	BCL	binomial	seed ~ FW * PH + SA + LA + LL	6	-	-341.26	180.63
M. laciniatus	BCG	binomial	sample size too small	1	-	-	-
M. laciniatus	F1	binomial	sample size too small	3	-	-	-
M. laciniatus	F2	binomial	sample size too small	1	-	-	-
M. laciniatus	hybrid	binomial	seed ~ PH + LA	11	2.86/1.34	-	-
M. laciniatus	all	binomial	seed ~ PH + LA	18	3.2/0.95	27.6	-7.8

Table S6. Model parameters for phenotypic selection analyses in 2021. Standard error (SE) for each trait is listed in order of the traits in the model.

Habitat	Geno	Model	n	SE	AIC	Log-L
M. guttatus	HG	poisson	39	0.28	302.4	-147.2
M. guttatus	HL	poisson	66	0.233	552.9	-272.4
M. guttatus	BCL	poisson	41	0.41	299.3	-145.7
M. guttatus	BCG	poisson	27	0.3	189.2	-90.6
M. guttatus	F1	poisson	48	0.31	387	-189.5
M. guttatus	F2	poisson	19	0.36	128.3	-60.1
M. guttatus	LL	poisson	4	0.93	34.8	-13.4
M. guttatus	hybrid	poisson	135	0.17	999.3	-495.7
M. guttatus	all	poisson	245	0.12	1884.5	-938.3
Hybrid	HG	poisson	8	0.14	68.2	-30.1
Hybrid	HL	poisson	18	0.34	136.4	-64.2
Hybrid	BCL	poisson	19	0.4	131.5	-61.8
Hybrid	BCG	poisson	11	0.79	87.6	-39.8
Hybrid	F1	poisson	33	0.38	238.3	-115.1
Hybrid	F2	poisson	9	1.9	62	-27
Hybrid	LL	poisson	-	-	-	-
Hybrid	hybrid	poisson	72	0.26	502.8	-247.4

Hybrid	all	poisson	102	0.18	735.9	-363.9
M. laciniatus	HG	poisson	18	0.65	141.2	-66.6
M. laciniatus	HL	poisson	31	0.39	234.7	-113.3
M. laciniatus	BCL	poisson	34	0.46	230.5	-111.3
M. laciniatus	BCG	poisson	29	0.46	204	-98
M. laciniatus	F1	poisson	78	0.25	615.8	-303.9
M. laciniatus	F2	poisson	33	0.56	256.1	-124
M. laciniatus	LL	poisson	27	0.41	187.2	-89.6
M. laciniatus	hybrid	poisson	174	0.04	3094	-1545
M. laciniatus	all	poisson	251	0.13	1861.8	-926.9
M. guttatus	HG	binomial	49	0.26	105.7	-47.8
M. guttatus	HL	binomial	72	0.22	153.2	-71.6
M. guttatus	BCL	binomial	49	0.35	105.9	-47.9
M. guttatus	BCG	binomial	34	0.44	-	-
M. guttatus	F1	binomial	53	0.3	115.5	-52.7
M. guttatus	F2	binomial	30	0.32	65.2	-27.6
M. guttatus	LL	binomial	-	-	-	-
M. guttatus	hybrid	binomial	166	0.15	-	-
M. guttatus	all	binomial	293	0.1	-	-
Hybrid	HG	binomial	10	0.47	29.3	-9.6
Hybrid	HL	binomial	22	0.47	53.2	-21.6
Hybrid	BCL	binomial	31	0.32	-	-
Hybrid	BCG	binomial	17	0.99	40.4	-15.2
Hybrid	F1	binomial	42	0.24	91.5	-40.7
Hybrid	F2	binomial	17	0.65	39.2	-14.6
Hybrid	LL	binomial	-	-	-	-
Hybrid	hybrid	binomial	107	0.17	-	-
Hybrid	all	binomial	147	0.14	287.3	-138.7
M. laciniatus	HG	binomial	30	0.5	63.9	-26.9
M. laciniatus	HL	binomial	46	0.29	96.2	-43.1
M. laciniatus	BCL	binomial	50	0.42	-	-
M. laciniatus	BCG	binomial	41	0.32	86	-38
M. laciniatus	F1	binomial	96	0.29	197.4	-93.7
M. laciniatus	F2	binomial	45	0.38	96.3	-43.1
M. laciniatus	LL	binomial	33	0.51	-	-
M. laciniatus	hybrid	binomial	232	0.16	455.3	-222.7
M. laciniatus	all	binomial	342	0.12	662.2	-326.1

Table S7. Model parameters for phenotypic selection analyses in 2023 on flowering time. Standard error (SE) for each trait is listed in order of the traits in the model.
